# Cemented versus uncemented hemiarthroplasty for femoral neck fracture in patients aged 75 years or older: A retrospective cohort study with 3-year follow-up

**DOI:** 10.1051/sicotj/2026080

**Published:** 2026-10-07

**Authors:** Daniel Donici, Youssef Jamaleddine, Emeline Chapron, Rémy Coulomb, Pascal Kouyoumdjian

**Affiliations:** 1 Orthopedic and Trauma Surgery Department, Hospital and University Center of Caremeau Nîmes. 4 Rue du Professeur Robert Debré 30900 Nîmes France; 2 Department of Orthopedic Surgery, Lebanese American University Medical Center-Rizk Hospital Beirut Lebanon; 3 Université Montpellier 1, 2 Rue de l'École de Médecine 34090 Montpellier France; 4 Laboratoire de Mécanique et Génie Civile (LMGC) CNRS-UM1 860 Rue de St–Priest 34090 Montpellier France

**Keywords:** Femoral neck fracture, Hemiarthroplasty, Cemented stem, Uncemented stem, Mortality and complications

## Abstract

*Introduction*: Optimal femoral stem fixation for hemiarthroplasty in older adults with displaced intracapsular fractures remains controversial. Cemented stems may reduce mechanical failure, but cementation carries risks. This study compared mortality and complications after cemented versus uncemented hemiarthroplasty in patients aged 75 years or older. *Methods*: We performed a retrospective study of consecutive patients aged 75 years or older who underwent hemiarthroplasty for intracapsular fracture between January 2015 and April 2022. Patients were grouped by stem fixation and followed until death or censoring in December 2025, allowing at least three years of follow-up. Outcomes included periprosthetic femoral fracture, dislocation, periprosthetic joint infection, a composite of any complication, and all-cause mortality from the early postoperative period to 3 years. Multivariable Cox regression was used for 3-year mortality, and a Fine-Gray competing risk model, with death as the competing event, was used for 3-year complications. *Results*: We included 531 patients (266 cemented, 265 uncemented). Periprosthetic femoral fracture was more frequent with uncemented stems (4.5% vs. 1.5%, *p* = 0.042), whereas dislocation was more frequent with cemented stems (6.4% vs. 2.6%, *p* = 0.038). Periprosthetic joint infection and any complication did not differ significantly between groups at 1 or 3 years, and mortality did not differ at any assessed time point (all *p* > 0.05). Cemented fixation was not independently associated with 3-year mortality (HR 0.84, 95% CI 0.66–1.07; *p* = 0.166) or 3-year any complication (sHR 1.01, 95% CI 0.59–1.74; *p* = 0.977). Older age, male sex, and ASA class 3 to 4 independently predicted higher 3-year mortality. *Discussion*: In patients aged 75 years or older, stem fixation was associated with different complication patterns, with more periprosthetic fractures after uncemented fixation and more dislocations after cemented fixation. Despite these differences in specific complications, cemented fixation was not independently associated with 3-year mortality.

## Introduction

Hip fracture is a major public-health problem in the elderly, frequently resulting in abrupt loss of independence and substantial excess mortality [1]. Although 1-year mortality has traditionally been quoted at approximately 30%, a recent systematic review indicates that outcomes have improved over time and now it is around 22% [2]. Hemiarthroplasty is the standard treatment for displaced femoral neck fractures in frail geriatric patients with low functional demands. However, its use in healthy, active elderly patients remains controversial [3]. Evidence-based guidance recommends cemented femoral stems when arthroplasty is performed in the elderly for femoral neck fracture [4].

The choice between cemented and uncemented fixation remains controversial in hemiarthroplasty. A multicentre randomized trial has shown that cemented fixation provides modest improvements in health-related quality of life and reduces the risk of periprosthetic fracture [5]. A meta-analysis of nine randomized trials similarly found substantially fewer periprosthetic fractures with cemented stems and no difference in perioperative mortality [6]. A large observational cohort study has demonstrated an association between uncemented fixation and an increased risk of aseptic revision [7].

However, cementation introduces the risk of bone cement implantation syndrome (BCIS), characterized by hypoxia, hypotension, and cardiovascular collapse during cementation [8]. In the setting of hemiarthroplasty, BCIS is a common complication, and severe BCIS has been associated with a markedly increased early mortality [9]. In addition, compared with uncemented hemiarthroplasty, cemented hemiarthroplasty is associated with higher mortality in the first 48 h postoperatively, with no differences at 7 days, 30 days, or 1 year [10].

In view of the clinically relevant balance between mechanical failure and cement-related risk, the primary hypothesis of this study was that cemented hemiarthroplasty would not increase all-cause mortality compared with uncemented hemiarthroplasty in patients aged ≥75 years with intracapsular femoral neck fracture. Secondary hypotheses were that the stem fixation method would influence the incidence of major postoperative complications, including periprosthetic femoral fracture, dislocation, and periprosthetic joint infection. To test these hypotheses, we compared outcomes between cemented and uncemented hemiarthroplasty over a 3-year follow-up.

## Materials and methods

### Study design and population

This single-center retrospective comparative cohort study evaluated outcomes after hemiarthroplasty performed for intracapsular femoral neck fractures in older patients. Patients were classified according to femoral stem fixation (cemented vs. uncemented).

Consecutive patients aged 75 years or older who underwent primary hemiarthroplasty for an intracapsular femoral neck fracture between 1 January 2015 and 6 April 2022 were eligible. We followed patients from the date of the index hemiarthroplasty until death or administrative censoring on 16 December 2025 (end of follow-up), ensuring a minimum potential follow-up of 3 years. Total hip arthroplasty cases were excluded in the main analyses.

### Surgical technique

All procedures were performed using conventional hemiarthroplasty techniques routinely employed in our institution, without computer navigation, robotic assistance, or patient-specific instrumentation. The posterolateral (Moore) approach was used in most cases, with an anterior (Hueter) approach used in a minority. With the Moore approach, capsular closure and posterior soft-tissue repair were routinely performed. All hemiarthroplasties were bipolar, with 22.2- or 28-mm inner femoral heads and variable neck lengths.

Femoral stem fixation was achieved using either a cemented or an uncemented press-fit stem. Uncemented fixation relied on an anatomical modular MBA stem, whereas cemented fixation used a PAVI cemented stem, both manufactured by Lépine (France). Cemented stems were implanted using third-generation cementation techniques. The fixation strategy mainly reflected two successive institutional periods with different departmental preferences. Uncemented fixation was routinely used during the earlier period, whereas cemented fixation became standard thereafter to reduce periprosthetic fracture risk. No predefined bone-quality criterion guided fixation choice.

Postoperative thromboprophylaxis consisted of prophylactic enoxaparin 40 mg once daily for 35 days unless therapeutic anticoagulation was indicated.

### Data collection

Demographics and baseline characteristics included age at surgery, sex, body mass index (BMI), ASA class, surgical approach, and documented cognitive impairment or dementia. Available radiographic variables included Dorr classification, Singh index, cortical bone ratio, and radiographic loosening.

The outcomes of interest were postoperative periprosthetic femoral fracture, hip dislocation, periprosthetic joint infection (PJI), a composite “any complication” endpoint, and all-cause mortality. Postoperative periprosthetic femoral fracture was defined as a femoral fracture around or distal to the stem after the index procedure, excluding intraoperative fractures, and was classified according to the Vancouver classification [11]. Dislocation was defined as radiographically confirmed displacement of the prosthetic head requiring reduction. PJI was defined according to the 2018 International Consensus Meeting (ICM) criteria [12]. Dislocation recurrence and treatment were recorded from the medical record.

The composite “any complication” endpoint was defined as the occurrence of at least one of the following events: periprosthetic femoral fracture, hip dislocation, or PJI. Patients could experience more than one type of complication; accordingly, counts for individual complication categories were not mutually exclusive, whereas each patient contributed only once to the composite endpoint, irrespective of the number of events experienced.

Vital status was available for all included patients. Mortality was assessed at 24 h, 48 h, 7 days, 30 days, 90 days, 1 year, and 3 years after surgery. For the 1- and 3-year outcome analyses, administrative censoring was applied at 365 and 1095 days, respectively, regardless of longer individual follow-up.

### Data analysis

Continuous variables are presented as mean ± standard deviation (SD) and were compared between groups using Welch’s *t*-test. Categorical variables are presented as counts (percentages) and were compared using Pearson’s chi-square test or Fisher’s exact test, as appropriate. All tests were two-sided, with statistical significance set at *p* < 0.05. Ninety-five percent confidence intervals for event proportions were calculated using the Wilson score method.

Two multivariable time-to-event models were fitted. Three-year all-cause mortality was analyzed using a Cox proportional hazards model, with results reported as hazard ratios (HRs) and 95% confidence intervals (CIs). For the 3-year “any complication” endpoint, death was treated as a competing event and a Fine-Gray subdistribution hazards model was applied; results are presented as subdistribution hazard ratios (sHRs) with 95% CIs.

Both models included stem fixation (cemented vs. uncemented), ASA class (3–4 vs. 1–2), age (per year), sex (male vs. female), BMI, and documented cognitive impairment or dementia as covariates. Statistical analyses were performed using IBM SPSS Statistics, version 27.0 (IBM Corp., Armonk, NY, USA). Fine-Gray competing-risk regression was implemented through the STATS COMPRISK extension.

## Results

This study included 531 patients. Baseline characteristics of the patients, stratified by femoral stem fixation (cemented vs. uncemented), are summarized in Table 1.

**Table 1:** Baseline characteristics by stem fixation type.

Variable		Cemented (*n* = 266)	Uncemented (*n* = 265)	*p*-value
Age at surgery (years), mean ± SD		88.1 ± 5.3	86.4 ± 5.8	<0.001
Sex	Male	61 (22.9%)	67 (25.3%)	0.527
	Female	205 (77.1%)	198 (74.7%)	
BMI (kg/m^2^), mean ± SD		23.2 ± 4.6	23.1 ± 4.3	0.978
Approach	Moore	252 (94.7%)	265 (100.0%)	<0.001
	Hueter	14 (5.3%)	0 (0.0%)	
ASA class	1–2	67 (25.2%)	92 (34.7%)	0.017
	3–4	199 (74.8%)	173 (65.3%)	
Cognitive impairment or dementia	No	122 (45.9%)	138 (52.1%)	0.152
	Yes	144 (54.1%)	127 (47.9%)	

Dorr classification did not differ between groups (*p* = 0.609). Mean cortical bone ratio was higher in the cemented group (0.565 ± 0.086 vs. 0.527 ± 0.086, *p* < 0.001), while Singh index distributions were similar. Radiographic loosening was identified in one uncemented case.

Dislocation occurred more frequently in the cemented group (17 [6.4%] vs. 7 [2.6%]), *p* = 0.038). A representative radiographic sequence of a cemented PAVI hemiarthroplasty complicated by dislocation is shown in Figure 1. Periprosthetic femoral fracture occurred more frequently with uncemented stems (12 [4.5%] vs. 4 [1.5%], *p* = 0.042). A representative radiographic sequence of an uncemented MBA hemiarthroplasty complicated by periprosthetic femoral fracture is shown in Figure 2. All dislocations occurred after the Moore approach. Cognitive impairment or dementia was documented in 16 of 24 patients with dislocation (12/17 cemented and 4/7 uncemented). Among cemented dislocations, 16/17 involved a 28-mm inner head and 12/17 a neutral neck length, compared with 7/7 and 6/7, respectively, in the uncemented group. Recurrent dislocation occurred in 7/17 cemented and 4/7 uncemented cases. Closed reduction alone was definitive in 9/17 cemented and 1/7 uncemented cases, whereas operative treatment was required in 8/17 and 6/7 cases, respectively. Within 3 years, reoperation was required in 18/266 (6.8%) cemented and 26/265 (9.8%) uncemented cases, while revision arthroplasty was performed in 9/266 (3.4%) and 19/265 (7.2%), respectively (Table 2). Revisions were mainly performed for dislocation in the cemented group and periprosthetic fracture in the uncemented group. Rates of periprosthetic joint infection and any complication did not differ significantly between groups. Mortality was also comparable at 24 h, 48 h, 7 days, 30 days, 90 days, 1 year, and 3 years (all *p* > 0.05) (Table 2).

**Figure 1:**
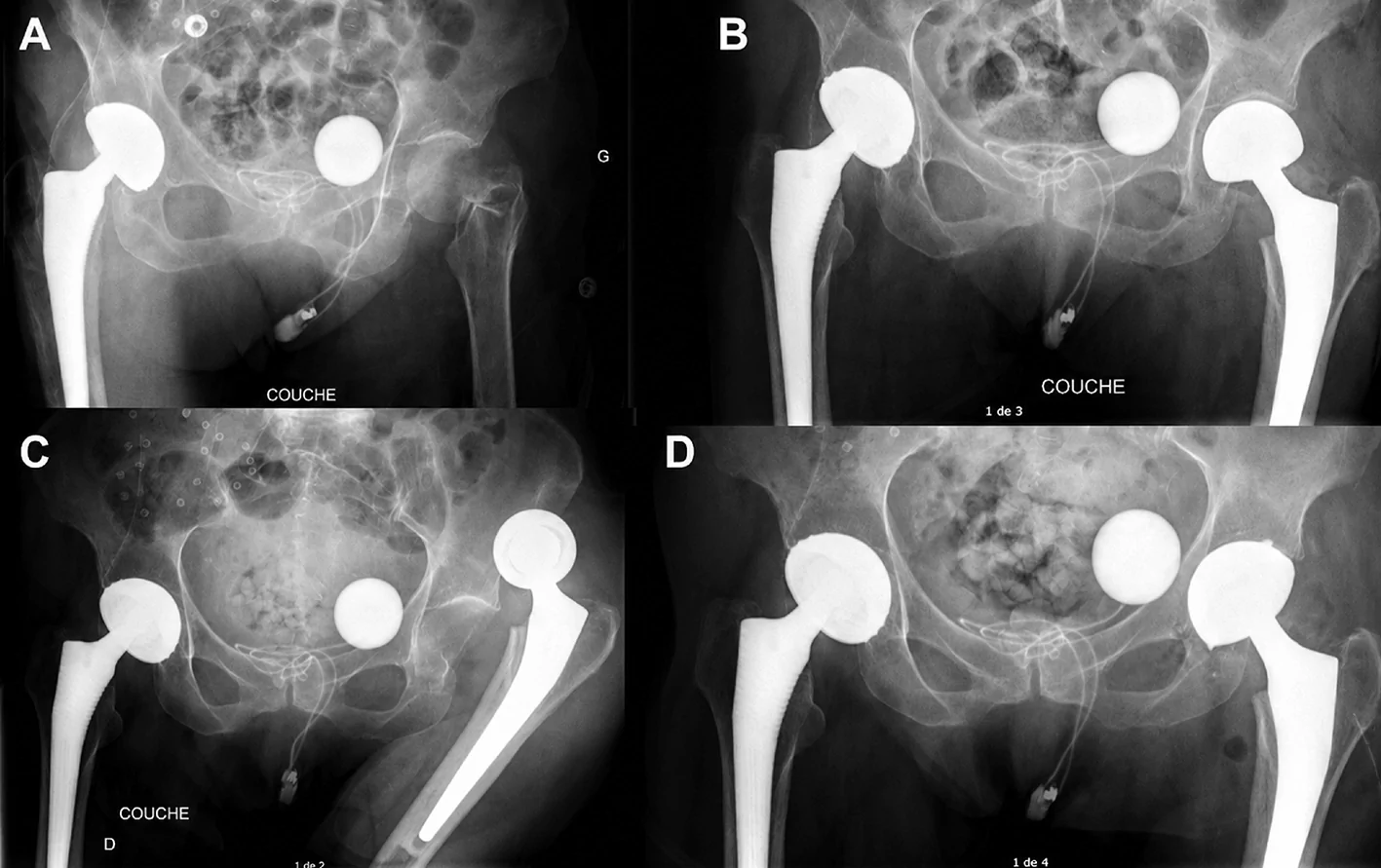
Representative radiographic sequence of a cemented PAVI hemiarthroplasty complicated by dislocation. (A) Preoperative anteroposterior pelvic radiograph showing a displaced intracapsular femoral neck fracture. (B) Postoperative anteroposterior pelvic radiograph after cemented PAVI hemiarthroplasty. (C) Subsequent radiograph demonstrating dislocation of the hemiarthroplasty. (D) Radiograph after reduction of the dislocation.

**Figure 2:**
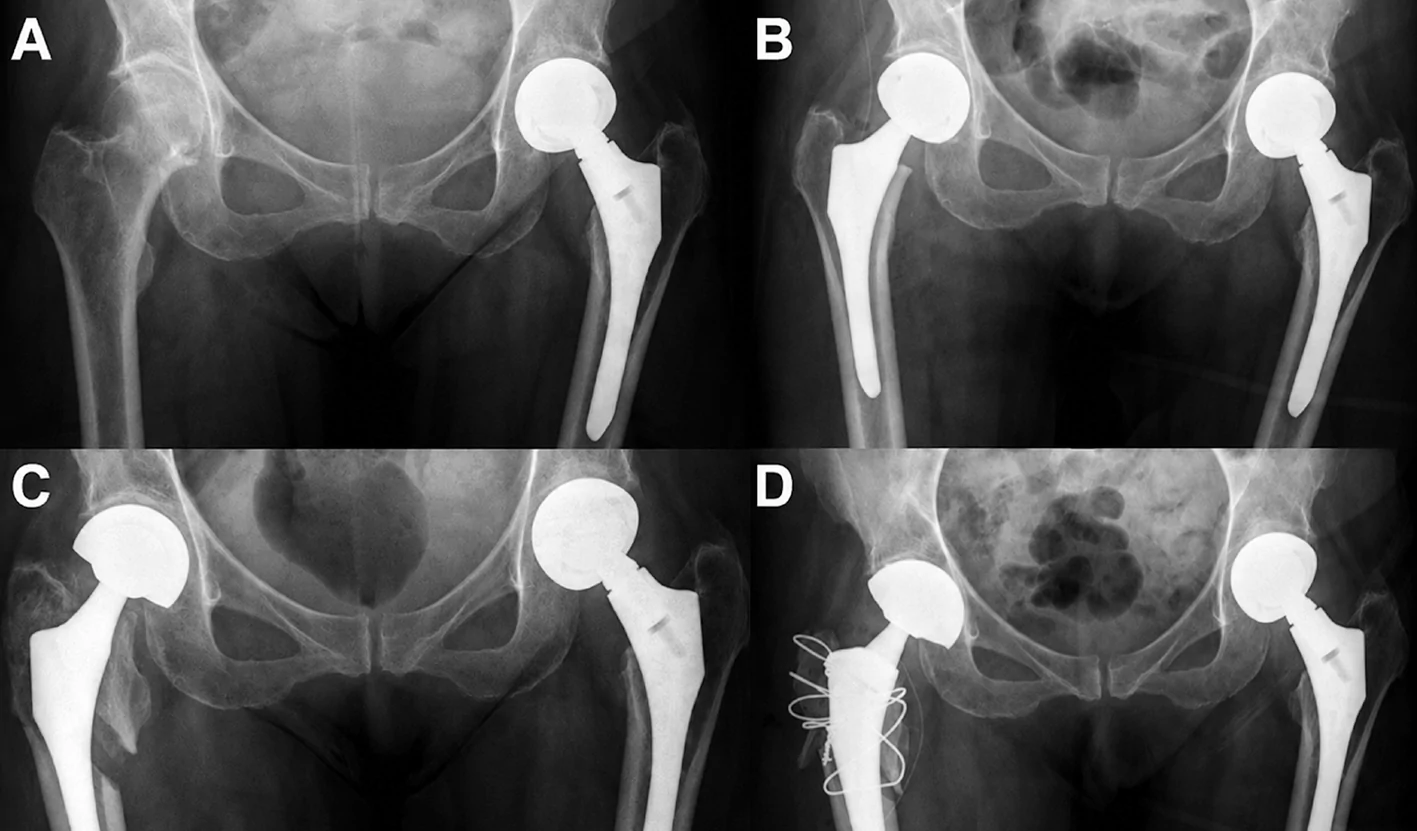
Representative radiographic sequence of an uncemented MBA hemiarthroplasty complicated by periprosthetic femoral fracture. (A) Preoperative anteroposterior pelvic radiograph showing an intracapsular femoral neck fracture. (B) Postoperative anteroposterior pelvic radiograph after uncemented MBA hemiarthroplasty. (C) Subsequent radiograph demonstrating a periprosthetic femoral fracture around the MBA stem. (D) Postoperative radiograph after revision with a long femoral stem and cerclage fixation.

**Table 2:** Postoperative outcomes. Complication subtypes were not mutually exclusive; “any complication” counts unique patients with ≥1 event (for example: complications in uncemented patients within 1 year: 26 events in 25 unique patients).

Outcome	Cemented	Uncemented	*p*-value
Periprosthetic femoral fracture within 1 year	4 (1.5%; 0.6–3.8)	12 (4.5%; 2.6–7.7)	0.042
Periprosthetic femoral fracture within 3 years	4 (1.5%; 0.6–3.8)	12 (4.5%; 2.6–7.7)	0.042
Dislocation within 1 year	17 (6.4%; 4.0–10.0)	7 (2.6%; 1.3–5.4)	0.038
Dislocation within 3 years	17 (6.4%; 4.0–10.0)	7 (2.6%; 1.3–5.4)	0.038
Periprosthetic joint infection within 1 year	8 (3.0%; 1.5–5.8)	7 (2.6%; 1.3–5.4)	0.799
Periprosthetic joint infection within 3 years	8 (3.0%; 1.5–5.8)	9 (3.4%; 1.8–6.3)	0.799
Any complication within 1 year	27 (10.2%; 7.1–14.4)	25 (9.4%; 6.5–13.6)	0.781
Any complication within 3 years	27 (10.2%; 7.1–14.4)	27 (10.2%; 7.1–14.4)	0.988
Mortality within 24 h	1 (0.4%; 0.1–2.1)	2 (0.8%; 0.2–2.7)	0.624
Mortality within 48 h	1 (0.4%; 0.1–2.1)	2 (0.8%; 0.2–2.7)	0.624
Mortality within 7 days	3 (1.1%; 0.4–3.3)	4 (1.5%; 0.6–3.8)	0.724
Mortality within 30 days	16 (6.0%; 3.7–9.5)	18 (6.8%; 4.3–10.5)	0.714
Mortality within 90 days	35 (13.2%; 9.6–17.7)	32 (12.1%; 8.7–16.5)	0.707
Mortality within 1 year	67 (25.2%; 20.3–30.7)	63 (23.8%; 19.0–29.3)	0.705
Mortality within 3 years	134 (50.4%; 44.4–56.3)	139 (52.5%; 46.4–58.4)	0.632
Any reoperation within 3 years	18 (6.8%; 4.3–10.4)	26 (9.8%; 6.8–14.0)	0.203
Any revision arthroplasty within 3 years	9 (3.4%; 1.8–6.3)	19 (7.2%; 4.6–10.9)	0.051

Values are *n* (%; 95% CI).

In multivariable Cox analysis (Table 3), cemented fixation was not independently associated with 3-year mortality (HR 0.84, 95% CI 0.66–1.07; *p* = 0.166). Higher ASA class, older age, and male sex remained independently associated with mortality, whereas BMI and cognitive impairment or dementia were not.

**Table 3:** Multivariable analysis for 3-year mortality and 3-year any complication.

Covariate	3-year mortality hazard ratio (95% CI)	*p*-value	3-year any complication subdistribution hazard ratio (95% CI)	*p*-value
Cemented stem (vs. uncemented)	0.84 (0.66–1.07)	0.166	1.01 (0.59–1.74)	0.977
ASA 3–4 (vs. 1–2)	2.64 (1.89–3.69)	<0.001	0.95 (0.52–1.72)	0.853
Age at surgery (per year)	1.05 (1.02–1.07)	<0.001	0.99 (0.95–1.04)	0.799
Male sex (vs. female)	2.17 (1.68–2.81)	<0.001	1.60 (0.89–2.86)	0.115
BMI (per kg/m^2^)	0.99 (0.97–1.02)	0.600	1.01 (0.95–1.07)	0.820
Cognitive impairment/dementia (vs. none)	1.21 (0.95–1.54)	0.117	1.22 (0.71–2.10)	0.473

In the Fine-Gray model, cemented fixation was not associated with 3-year any complication (sHR 1.01, 95% CI 0.59–1.74; *p* = 0.977).

## Discussion

In this retrospective cohort of 531 consecutive patients aged ≥75 years undergoing hemiarthroplasty for intracapsular femoral neck fracture, uncemented stems were associated with a higher rate of periprosthetic femoral fracture (4.5% vs. 1.5%), whereas cemented stems were associated with a higher rate of hip dislocation (6.4% vs. 2.6%). Rates of PJI and overall complications at 1 and 3 years were not significantly different between groups, while mortality was comparable at all assessed time points from 24 h to 3 years. Stem fixation type was not independently associated with 3-year mortality. In the Fine-Gray competing-risk analysis for 3-year “any complication,” none of the covariates, including stem fixation, ASA class, age, sex, BMI, or cognitive impairment or dementia, were independently associated with complications. After adjustment for age, sex, ASA class, BMI, and cognitive impairment or dementia, stem fixation was not independently associated with 3-year mortality, while older age, male sex, and higher ASA class remained independent predictors.

The higher periprosthetic fracture rate with uncemented fixation in our cohort is concordant with the available comparative literature. In a multicentre randomized trial, cemented hemiarthroplasty resulted in fewer periprosthetic femoral fractures compared with uncemented hemiarthroplasty [5]. Similarly, a recent level-I meta-analysis of randomized trials reported lower periprosthetic fracture risk with cemented stems [6]. Longer-term randomized follow-up has also raised concern that uncemented stems may carry an “unacceptably high” risk of later femoral fracture in elderly patients [13]. From a clinical standpoint, this complication is highly relevant in patients aged ≥75 years because periprosthetic fracture frequently necessitates reoperation, often leading to prolonged immobility, loss of function, and increased physiological stress.

We observed a higher dislocation rate after cemented hemiarthroplasty. All dislocations occurred after the Moore approach despite routine posterior soft-tissue repair, and most involved a 28-mm inner head and neutral neck length. Cognitive impairment or dementia was frequent among patients with dislocation. In a national cohort from the Swedish Hip Arthroplasty Register, dislocation after hemiarthroplasty was common, and the strongest risk factor was the posterior approach; dementia was also an independent risk factor [14]. Another study similarly demonstrated a higher dislocation risk with the posterolateral approach compared with anterolateral approaches, with posterior repair reducing this excess risk [15]. However, the available clinical and implant characteristics do not fully explain the higher rate observed with cemented fixation. Residual confounding and unmeasured factors, including component positioning, offset restoration, and postoperative behavior, remain possible; therefore, this difference cannot be attributed to fixation type alone. Additionally, the cemented fixation permits considerable technique-dependent variation in femoral stem anteversion, whereas anatomical uncemented stems more closely reproduce native femoral version and therefore may limit variability.

We used a Fine-Gray competing-risk model to assess the association between covariates and the cumulative incidence of complications, accounting for death as a competing event, in line with recommended practices for transparent reporting and interpretation of subdistribution hazard ratios [16]. Within this model, no covariate was independently associated with the cumulative incidence of complications. Because the composite endpoint combines complications with distinct etiologies and risk profiles, its use may dilute covariate-specific effects, particularly in a population with high competing mortality. Accordingly, stem fixation may differentially affect specific complications, including periprosthetic fracture and dislocation, while having no measurable impact on the overall complication burden in this high-mortality population.

We observed no significant differences in mortality between fixation groups at any assessed time point from the early postoperative period through 3 years. In multivariable analysis, fixation was not independently associated with 3-year mortality. This is consistent with a randomized trial study showing no increase in 12-month mortality with cemented hemiarthroplasty [5]. However, the cement debate is strongly influenced by concern for bone cement implantation syndrome (BCIS), which can manifest as peri-cementation hypoxia, hypotension, arrhythmia, and cardiovascular collapse [8]. Severe BCIS is uncommon but is associated with markedly increased early mortality in cemented hemiarthroplasty [9]. Patient-safety surveillance has also documented rare perioperative deaths or severe harm temporally linked to cement insertion during hemiarthroplasty for fractured neck of femur [17]. In addition, compared with uncemented hemiarthroplasty, cemented hemiarthroplasty is associated with higher mortality in the first 48 h postoperatively, with no differences at 7 days, 30 days, or 1 year [10]. Taken together, these findings suggest that the principal risk-benefit balance of cementation lies in the immediate intraoperative and early postoperative period (within the first 48 h), whereas any differences in outcomes tend to attenuate with longer-term follow-up. In this context, adherence to perioperative mitigation strategies, emphasizing team communication at the time of cementation, hemodynamic optimization, and technical measures to limit intramedullary pressure, remains essential [18].

The independent associations of older age, male sex, and higher ASA class with mortality in our cohort are consistent with previous studies. A meta-analysis demonstrated substantial excess mortality after hip fracture that persists over time and is consistently higher in males than in females [19]. Another study reported higher mortality rates after hip arthroplasty among patients with higher ASA classifications [20]. Higher age has also been identified as an independent predictor of long-term mortality; for example, a population-registry study of patients managed with hemiarthroplasty [21].

This study has many strengths, which include consecutive case inclusion, complete ascertainment of vital status, and use of competing-risk modeling for non-fatal complications in a cohort with high mortality and long follow-up period. This study has several limitations inherent to its retrospective, non-randomised design. Fixation choice was not randomised, so residual confounding cannot be fully excluded. A period effect related to these successive institutional practices cannot be excluded. Information on pre-fracture functional level, delirium, and postoperative restrictions was not systematically available, despite their known influence on instability and postoperative outcomes. Although Dorr classification, Singh index, cortical bone ratio, and radiographic loosening were available, implant-positioning parameters such as stem version, offset restoration, leg length, cement mantle quality, subsidence, and canal fill were not systematically measured, limiting assessment of their contribution to dislocation risk.

Furthermore, the uncemented stems used in this series were anatomical, canal-filling implants rather than straight tapered stems, which may limit the generalisability of our findings to other uncemented designs. Finally, only 54 patients experienced the 3-year composite complication endpoint, and six covariates were included in the Fine-Gray model; therefore, the analysis may have been underpowered and at risk of overfitting, and adjusted complication estimates should be interpreted cautiously.

## Conclusion

In this cohort of 531 patients aged ≥75 years undergoing hemiarthroplasty for intracapsular femoral neck fracture, stem fixation was associated with different complication patterns. Periprosthetic femoral fractures occurred more frequently after uncemented fixation, whereas dislocations were more common with cemented stems. Periprosthetic joint infection and mortality were comparable between groups. In adjusted analyses, cementation was not independently associated with 3-year mortality or the cumulative incidence of complications. Older age, male sex, and higher ASA class remained independent predictors of mortality, while no modeled covariate was independently associated with the composite complication endpoint.

## Data Availability

The datasets generated and analyzed during the current study are not publicly available due to patient privacy.
